# Time-of-Day-Dependent Gating of the Liver-Spinal Axis Initiates an Anti-Inflammatory Reflex in the Rat

**DOI:** 10.1523/ENEURO.0463-20.2020

**Published:** 2020-12-10

**Authors:** Eva Soto-Tinoco, Esteban Santacruz, María del Carmen Basualdo-Sigales, Natalí N. Guerrero-Vargas, Ruud M. Buijs

**Affiliations:** 1 Departamento de Biología Celular y Fisiología, Instituto de Investigaciones Biomédicas, Universidad Nacional Autónoma de México, Ciudad de México 04510, México; 2 Departamento de Anatomía, Facultad de Medicina, Universidad Nacional Autónoma de México, Ciudad de México 04510, México

**Keywords:** circadian, inflammation, liver, reflex, spinal, sympathetic

## Abstract

The autonomic nervous system (ANS) modulates the immune response through the engagement of an anti-inflammatory reflex. There is controversy regarding which efferent branch of the ANS, sympathetic or parasympathetic, downregulates the intensity of the inflammatory response. Furthermore, how information about the immune status of the body reaches the CNS to engage this reflex remains unclear. The present study demonstrates the existence of a liver-spinal axis that conveys early circulating inflammatory information to the CNS in response to lipopolysaccharide (LPS) and serves as the afferent arm of a sympathetic anti-inflammatory reflex. Furthermore, brainstem and spinal cord visceral sensory neurons show a time-of-day-dependent sensitivity to the incoming inflammatory information, in particular, prostaglandins (PG). Consequentially, the liver-spinal axis promotes the retention of tumor necrosis factor α (TNFα) in the liver and spleen during the resting period, resulting in low plasmatic TNFα levels. Consistently, low sensitivity for LPS during the active period promotes the release of TNFα from the organs into the circulation, resulting in high plasmatic TNFα levels. The present novel findings illustrate how the time-of-day-dependent activation of the liver-spinal axis contributes to the daily fluctuations of the inflammatory response.

## Visual Abstract

**Figure:**
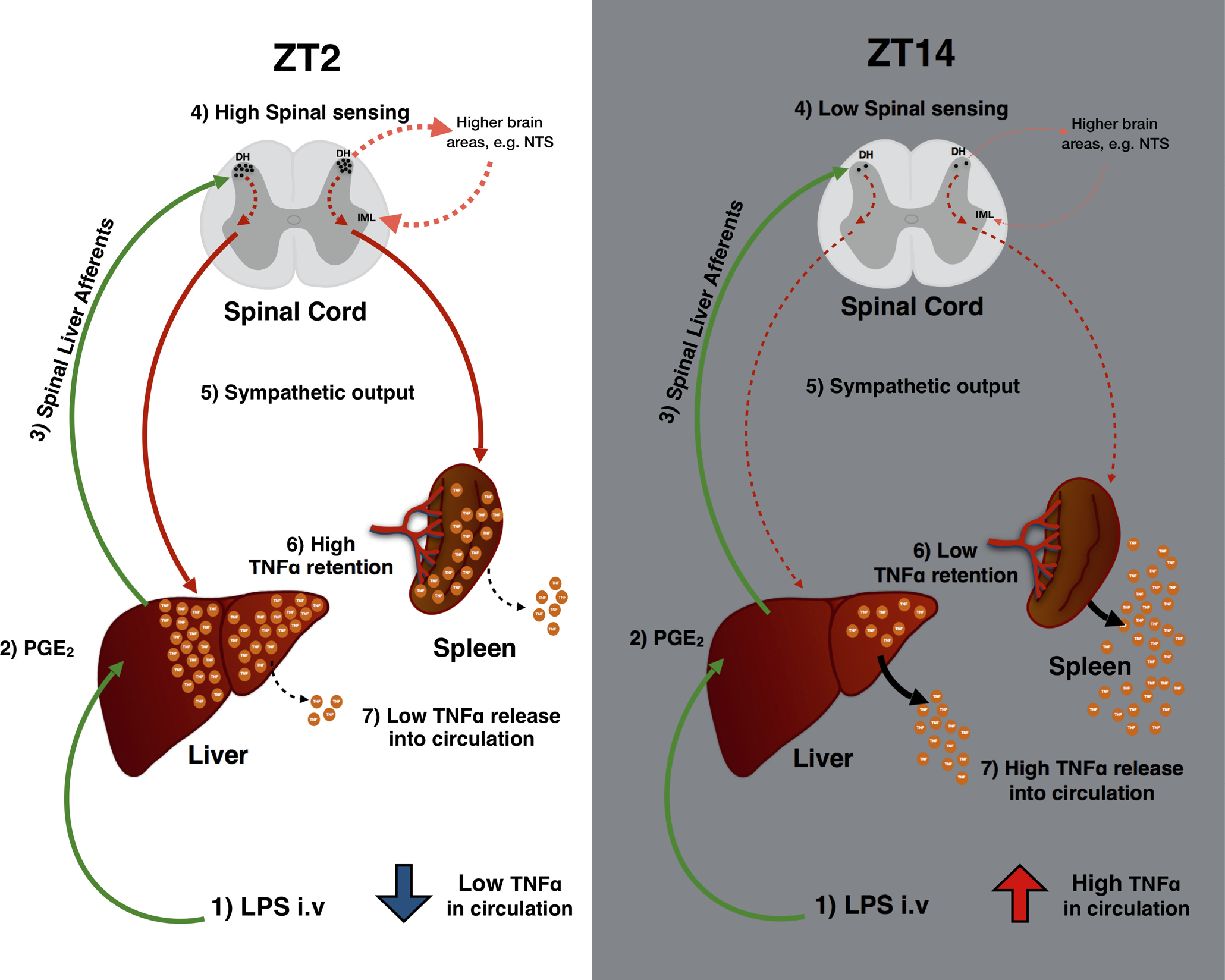

## Significance Statement

The anti-inflammatory reflex is a survival mechanism essential for homeostasis. An out-of-control inflammatory process is detrimental to the individual and it is the cornerstone of many chronic diseases. It has been suggested that the sensory vagus nerve works as the afferent arm of this reflex arc, detecting circulating cytokines to start the anti-inflammatory reflex. The present study provides strong evidence of an additional afferent arm, which we have named the liver-spinal axis, that senses early inflammatory mediators that precede cytokine release into circulation. As an additional layer of complexity, inflammation has adapted to the light/dark cycle. We demonstrate that the anti-inflammatory reflex is time-of-day-dependent, synchronizing the peak of the inflammatory response to when it is most likely needed.

## Introduction

The CNS regulates the immune system through the autonomic nervous system (ANS). Through its sympathetic and parasympathetic efferent branches, the ANS is proposed to modulate the intensity of inflammatory processes through autonomic reflexes (Borovikova et al., 2000; Martelli et al., 2014). Several areas of the CNS need to be informed about the presence of a circulating inflammatory stimulus, like lipopolysaccharide (LPS), to elicit an autonomic anti-inflammatory reflex, yet, it is unclear as to how inflammatory information reaches the CNS.

One possibility is that LPS directly enters the CNS, but evidence shows that LPS has a minimal penetration across the blood-brain barrier (BBB; Banks and Robinson, 2010).

Another possibility that has been explored is that cytokines inform the CNS about the immune status of the body (Dantzer, 2001; Zanos et al., 2018). However, cytokines are detectable in circulation 20–30 min after an LPS challenge, therefore, there is a critical time-window in which other mechanisms may be essential for the early signaling of inflammatory information to the CNS. Sensory structures in the CNS expressing Toll-like receptor 4 (TLR4), an LPS receptor, are possible candidates for the immediate sensing of LPS. These include the circumventricular organs (CVOs), specialized structures located near the cerebral ventricles characterized by their lack of BBB. The expression of TLR4 was indeed reported in the four sensory CVOs: the area postrema (AP), organum vasculosum of the lamina terminalis (OVLT), subfornical organ (SFO), and median eminence (Laflamme and Rivest, 2001; Wuchert et al., 2008; Ott et al., 2010; Nakano et al., 2015), making the CVOs ideal places for detecting circulating LPS. On the other hand, TLR4 is also expressed in visceral sensory neurons located in the nodose (vagal) and dorsal root (spinal) ganglia (Hosoi et al., 2005; Barajon et al., 2009), opening the possibility that visceral sensory neurons could detect the presence of circulating LPS. In this current study, we investigate the responsiveness of the CVOs and visceral sensory areas (VSAs) to an LPS challenge to uncover the pathways involved in the transmission of circulating inflammatory information to the CNS, as well as the ensuing response of the ANS. For the purposes of this study, we focused only on the events that take place within the first 50 min after the LPS administration. In particular, the response of tumor necrosis factor α (TNFα), one of the first proinflammatory cytokines to be released into circulation and a major promotor and modulator of the inflammatory process (Lukacs et al., 1995; Baud and Karin, 2001). This allowed us to have a robust parameter to measure the effects of our manipulations over the inflammatory response.

Furthermore, immune processes like cytokine release (Guerrero-Vargas et al., 2014), pathogen clearance (Tognini et al., 2017), leukocyte recruitment (Scheiermann et al., 2012), and lymphocyte egress from lymph nodes (Druzd et al., 2017) are all circadian driven. In particular, the TNFα response to LPS is stronger during the active period than during the resting period. Previous studies have demonstrated that the ANS imposes rhythmicity on various immune processes (Méndez-Ferrer et al., 2008; Scheiermann et al., 2012), opening the question of whether the anti-inflammatory reflex contributes to the rhythmicity of the TNFα response to LPS.

## Materials and Methods

### Animals and general housing conditions

Adult male Wistar rats weighing 280–300 g at the beginning of the experiments were obtained from the animal facility of the Faculty of Medicine of the Universidad Nacional Autónoma de México (UNAM). Animals were group housed until they reached the desired weight for experiments. After surgery, subjects were individually housed to avoid the disruption of the implanted jugular cannula because of social interaction. The animal facility had a 12/12 h light/dark cycle with lights on at 7 A.M., defined as zeitgeber time (ZT)0, and lights off at 7 P.M., defined as ZT12. The room was maintained at a regulated temperature ranging between 22°C and 25°C. All rats were given free access to water and food (Envigo Teklad 2018S). Experimental procedures used in this study were in strict accordance with the Mexican norms for animal handling (Norma Oficial Mexicana NOM-062-ZOO-1999), in agreement to international guidelines for animal handling, and approved by the Ethics Committee of the Instituto de Investigaciones Biomédicas, UNAM. All efforts were made to minimize the number of animals and their suffering.

### Reagents

LPS, *Escherichia coli* serotype 0127:B8, (Sigma-Aldrich, catalog #L4516, lot #051M4004) was reconstituted with sterile water. Recombinant rat TNFα (Shenandoah Biolotechnology Inc., catalog #P16599) was reconstituted with sterile water and diluted with a 0.1% BSA solution for prolonged storage. Indomethacin (Sigma-Aldrich, catalog #I7378) was reconstituted with 0.1 mm Na_2_CO_3_. Prostaglandin E_2_ (PGE_2_; Cayman Chemical, catalog #14 010) was reconstituted with 95% ethanol. Propranolol hydrochloride (Sigma-Aldrich, catalog #P0884) was reconstituted in sterile water. All reagents were further diluted in 0.9% pyrogen-free sterile saline to yield the desired concentration for their administration to the animals.

### Surgical procedures

All surgeries were performed using aseptic procedures and under deep anesthesia using a combination of intramuscular ketamine (100 mg/kg Clorkétam 1000 Q-7090-099, Vetoquinol) and xylazine (10 mg/kg, Procin, PiSA, Q-7833-099) anesthesia.

### Intrajugular catheter insertion

All animals underwent the insertion of a polyethylene silicon tube catheter (0.025 in. i.d. and 0.047 in. o.d.; SILASTIC Laboratory tubing; Dow Corning Corp.) into the right external jugular vein. The catheter was filled with heparin (500 U/ml) as anti-coagulant and antibiotics (100 U/ml, penicillin/streptomycin, Gibco antibiotic-antimycotic (100×), catalog #15240062) to prevent infections. We used dental cement to secure the catheter on the top of the skull using three stainless-steel screws inserted into the skull. Through this jugular catheter, animals received the different reagents mentioned previously and also blood was withdrawn the day of the experiment. Animals were allowed to recover from this surgery for at least 7 d before performing the actual experiment.

### Telemetry transmitter implantation

To determine whether LPS elicits a sympathetic activation, heart rate was evaluated with a Stellar telemetry transmitter (TSE Systems). The device was implanted as instructed by the manufacturer in the abdominal aorta, just rostral to the iliac bifurcation. The transmitter was sutured to the abdominal wall. Positive and negative bio-potential leads for the ECG recording were passed out of the abdomen and sutured to the left caudal rib region and to the right pectoral muscle, respectively. All incisions were closed and the animals were allowed to recover for 10 d before performing the actual experiment.

### Peritoneal capsaicin deafferentation

To determine whether peritoneal afferent nerves play a role in the early signaling of inflammation to the CNS, we induced a peritoneal deafferentation with capsaicin. The procedure used has been described elsewhere (Bret-Dibat et al., 1997). In brief, previously cannulated animals received a total dose of 25 mg/kg capsaicin (Natural, Sigma-Aldrich catalog #360376) intraperitoneally, which was divided into a series of 10 injections over a 48-h period. On both days, rats were injected with ketamine (10 mg/300 g, i.v.) and xylazine (1 mg/300 g, i.v.) for anesthetic purposes and were then treated with five doses of capsaicin (2.5 mg/kg in 300 μl of vehicle (Veh): 10% ethanol, 10% Tween 80, and 80% injectable saline 0.9%). Each injection was separated from the next by a 90-min recovery period. Sham animals received the same volume of Veh as capsaicin-treated animals. Animals were allowed to recover for 12 d before performing the experiments.

### Hepatic sensory denervation

#### Spinal

To elucidate with greater precision the involvement of hepatic sensory nerves in the early signaling of inflammation to the CNS, we performed a specific liver denervation that involved mechanical denervation followed by the application of local capsaicin to remove spinal sensory nerves. The procedure has been described elsewhere (Kalsbeek et al., 2004). On the same surgery on which the jugular cannula was placed, a laparotomy was performed in the midline. The liver lobes were gently pushed up, and ligaments around the liver lobes were severed to free the bile duct and portal vein complex, which were isolated from each other. At the level of the hepatic portal vein, the hepatic artery breaks up into the hepatic artery proper and the gastroduodenal artery. This division occurs on the ventral surface of the portal vein. At this point, the arteries were separated via blunt dissection from the portal vein. Nerve bundles running along the hepatic artery proper were removed using microsurgical instruments under an operating microscope (25× magnification). Any connective tissue attachments between the hepatic artery and portal vein were also removed, eliminating any possible nerve crossings. After this procedure, 10 µl of capsaicin (50 mg/ml) was applied on top of the bile duct and portal vein/hepatic artery complex. This procedure has been described elsewhere (Fujita et al., 2007). Spreading of capsaicin to the peritoneum was prevented by isolating this complex with a saline-soaked cotton and by using a very small volume of the capsaicin solution. The capsaicin solution was let to act and dry for ∼10 min over the hepatic portal system and afterward, the peritoneum is closed. Animals were allowed to recover for 12 d before performing the experiments.

#### Vagal

To determine the role of the vagus nerve in the early signaling of inflammation to the CNS, we performed a hepatic vagotomy. Since it is known that the vagus is a mixed sensory-motor nerve, and depending on the species, ∼70–80% of the nerve comprises sensory fibers (Browning et al., 2017) this is a valid approach to remove the afferent vagus. The procedure has been described elsewhere (Kalsbeek et al., 2004). On the same surgery on which the jugular cannula was placed, a laparotomy was performed in the midline. The fascia containing the hepatic branch was stretched by gently moving the stomach and the esophagus. With a myelin-specific dye (Toluidin Blue), the hepatic branch could be revealed as it separates from the left vagal trunk. With the aid of a binocular-operating microscope, the stained neural tissue was transected between the ventral vagus trunk and liver. Also, small blue-stained branches running in the fascia between the stomach and the liver were transected. Afterwards, the peritoneum is closed.

As a control, we performed a sham surgery, in which a laparotomy was performed in the midline and the liver, and its nerves were manipulated, but not severed. Afterwards, the peritoneum is closed. Animals were allowed to recover for 12 d before performing the experiments.

### Sample collection and ELISA for TNFα and PGE_2_

In all experiments, blood samples were collected before (time 0 was used as an inside control) and 50 min after the respective treatment. Hereafter, animals were deeply anesthetized with an overdose of pentobarbital iv. Afterwards, the peritoneal cavity was opened, spleen and liver were collected fresh and the animal was then perfused with saline followed by 4% paraformaldehyde in PBS. TNFα levels in plasma, spleen and liver, were determined with an ELISA developed in our laboratory. Rat TNFα mouse recombinant monoclonal antibody (R&D Systems, catalog #MAB510R, RRID:AB_2876875) was used as the capture antibody and Rat TNFα goat polyclonal biotinylated antibody (R&D Systems, catalog #BAF510, RRID:AB_356538) was used as the detection antibody. Following the instructions of the manufacturer, PGE_2_ concentration in plasma was determined with a competitive ELISA that quantifies a primary metabolite of PGE_2_ since the latter is too unstable to be measured directly (Cayman Chemical, catalog #514531, RRID:AB_2876793).

### Immunohistochemistry

Following perfusion, spinal cord, brain and brainstem were removed, postfixed for 24 h, cryoprotected in 30% sucrose for 48–72 h, frozen, and cut in coronal sections of 40 μm at −20°C in a cryostat. Free floating sections were incubated with c-Fos rabbit polyclonal antibody (1:2000; Santa Cruz Biotechnology, catalog #sc-52) for 48 h at 4°C. Later, they were rinsed and incubated with a biotinylated donkey-anti rabbit antibody (1:500, Jackson ImmunoResearch, catalog #711-065-152, RRID**:** AB_2340593) and avidin–biotin complex (1:500, Vector Laboratories, catalog #PK-4000), for 2 h each at room temperature. Product visualization was obtained with 0.01% diaminobenzidine, 0.05% nickel ammonium sulfate and 0.01% hydrogen peroxide for 15 min. All sections were mounted, dehydrated, and cover-slipped with microscopy Entellan New (Merck/Sigma-Aldrich, catalog #107961).

### Quantification of c-Fos immunoreactivity

In order to quantify immunoreactivity of c-Fos in the DH, ten representative sections of thoracical (T7–T10) level sections were selected in accordance with the Atlas of the Rat Spinal Cord (Watson et al., 2009). For the nucleus of the solitary tract (NTS) and AP, 6 representative sections were selected (bregma −13.92, −14.16); for the SFO and OVLT, three representative sections were selected (bregma −0.72, −1.08 for the SFO and bregma 0.6, 0.36 for the OVLT), all in accordance with the *Atlas of the Rat Brain in Stereotactic Coordinates* (Paxinos and Watson, 2005). Images were examined under a light microscope and captured with a 20× ocular. An InfinityHD camera (Lumenera Software) was used to acquire all the images used in this study. Immunoreactive-positive nuclei were counted using a computerized image analysis system (ImageJ, 1.42q, National Institutes of Health) by determining the area of each nuclei by free hand. The background was subtracted and threshold was determined; particle analysis was set for particles of 1.0–2.0 circularity and 500–800 pixels. Background optic density was established in a nearby region lacking c-Fos immunoreactivity. The average of all the counts in the different sections, was taken as the final measurement.

### Western blot analysis

The procedure used has been described elsewhere (Iimuro et al., 1998); 5 mg of liver tissue was weighed and lysed with 300 µl of lysis buffer (50 mm Tris-HCl pH 8.0, 150 mm NaCl, 1% Triton X-100, 0.5% sodium deoxycholate, and 1% SDS) and 50 µl of protease inhibitor stock (Roche cOmplete Protease Inhibitor Cocktail, 11697498001, one tablet was diluted in 1 ml of PBS). The homogenization of the tissue was performed in a TissueLyser LT (QIAGEN). Samples were centrifuged at 14,000 rpm at 4°C for 10 min, and the supernatant was collected. Protein concentration was determined with a NanoDropTM 1000 Spectrophotometer (Thermo Fisher Scientific). Forty micrograms of protein from each sample was separated in SDS-PAGE and subsequently transferred to nitrocellulose membranes. The nitrocellulose membranes were rinsed and blocked with 3% BSA in TBS for 1 h at room temperature and cut in half at ∼60 kDa. The upper half was probed with a mouse monoclonal anti-TLR4 (1:4000, Novus Biologicals, catalog #NB100-56566, RRID:AB_2205129), and the lower half was probed with a mouse monoclonal anti-α-tubulin as loading control (1:5000, Sigma-Aldrich, catalog #T5168, RRID:AB_477579) for 24 h at 4°C. Later, both membranes were probed with a biotinylated donkey-anti mouse antibody (1:500, Jackson ImmunoResearch, catalog #715-065-150, RRID: AB_2307438) and avidin-biotin complex (1:500, Vector Laboratories, catalog #PK-4000) for 2 h each at room temperature. Immunoreactivities were visualized using 0.01% diaminobenzidine, 0.05% nickel ammonium sulfate, and 0.01% hydrogen peroxide for 5 min. Results were analyzed using ImageJ to measure the optic density of TLR4 immunoreactivity normalized with respect to the α-tubulin control levels in the same sample.

### Experimental design

During all experiments, animals were permanently connected to the blood-sampling line, which was attached to a metal collar and kept out of reach of the animals by means of a counterbalanced beam. This allowed all manipulations to be performed without handling the animals and maximally avoid a stress response. This remote manipulation also prevents the upregulation of c-Fos expression normally induced by the handling of the animals and observe only the c-Fos induced by the LPS challenge. The metal collars were attached 24 h before the actual experiment (Kalsbeek et al., 2012) to allow the animals to recover from the stress of the collar attachment.

#### Experiment 1: determine which area of the CNS responds early after an LPS challenge

Animals were cannulated and randomly assigned to one of two conditions, LPS (2 µg/kg) or Veh administration at ZT2 (*n* = 6/group). Blood samples were collected before and 50 min after the respective treatment for TNFα determination. Both groups were deeply anesthetized and transcardially perfused 50 min after the respective treatment. Brain, spinal cord and brainstem were processed for c-Fos immunohistochemistry.

#### Experiment 2: determine which molecule activates the CNS after an LPS challenge

Animals were cannulated and randomly assigned to one of five conditions: Veh (*n* = 6), LPS (2 µg/kg; *n* = 6), TNFα (5 µg/kg; *n* = 5), LPS (2 µg/kg) + indomethacin (10 mg/kg; *n* = 6) or PGE_2_ (100 µg/kg; *n* = 4) administration at ZT2. Blood samples were collected before and 50 min after the respective treatment for TNFα determination. Blood samples were collected before 10 and 20 min after LPS for PGE_2_ determination. All groups were deeply anesthetized and transcardially perfused 50 min after the respective treatment. Spinal cord and brainstem were processed for c-Fos immunohistochemistry. Additional animals for each group (Veh, LPS, LPS+Indo, *n* = 3/group) were implanted with a telemetry transmitter to measure heart rate, which was continuously monitored at 2.5-min intervals for 1 h.

#### Experiment 3: determine whether visceral sensory afferents detect LPS and start an anti-inflammatory reflex

Animals were cannulated and randomly assigned to one of two conditions: peritoneal deafferentation with capsaicin or sham (*n* = 5/group). Animals were allowed to recover for 12 d. Other series of animals were cannulated and randomly assigned to one of three conditions: sham, vagal sensory liver denervation (VLX), or spinal sensory liver denervation (SLX; *n* = 6/group). Animals were allowed to recover for 12 d. The day of the experiment, all animals were challenged with LPS (2 µg/kg) at ZT2. An additional set of animals received LPS or LPS+propranolol (1 mg/kg) at ZT2 (*n* = 5/group). Blood samples were collected before and 50 min after the respective treatment for TNFα determination. All animals were deeply anesthetized and transcardially perfused 50 min after their respective treatment. Spinal cord and brainstem were processed for c-Fos immunohistochemistry. Liver and spleen were processed for TNFα determination.

#### Experiment 4: determine whether the plasma TNFα rhythm is because of a time-of-day-dependent sensitivity to LPS in the VSAs

Animals were cannulated and randomly assigned to one of two conditions: LPS administration at ZT2 or LPS administration at ZT14 (2 µg/kg; *n* = 5/group). Another set of animals (*n* = 5/group) was kept in constant darkness from the night before the experiment started. In these experiments, time is indicated as circadian time (CT), indicating that the animal’s endogenous rhythm of ∼24 h is not entrained to external cues, in this case, light. These animals received LPS (2 µg/kg) at CT2 (2 h after the beginning of the subjective day) or CT14 (2 h after the beginning of the subjective night). Another set of animals was randomly assigned to one of three conditions: Veh, LPS, or LPS+indomethacin (10mg/kg) administration at ZT14 (*n* = 5–6/group). Another set of animals was randomly assigned to one of two conditions: low LPS (2 µg/kg) or high LPS (100 µg/kg) administration at ZT14 (*n* = 5/group). Blood samples were collected before and 50 min after the respective treatment for TNFα determination. Blood samples were collected before 10 and 20 min after LPS for PGE_2_ determination. All animals were deeply anesthetized and transcardially perfused 50 min after their respective treatment. Spinal cord and brainstem were processed for c-Fos immunohistochemistry. Liver and spleen were processed for TNFα determination. An additional set of animals (*n* = 3/group) was killed without any treatment at ZT2 or ZT14 to evaluate the basal TLR4 expression in the liver by Western blotting.

### Data analysis and statistics

Animals were randomly assigned to experimental time points or groups. Investigators were blinded to group allocation during data analysis. Animals that showed detectable levels of TNFα before the administration of LPS were excluded from the final analysis. Data analysis was performed using GraphPad Prism 6. All data were normally distributed and are expressed as mean ± SEM.

For the present study, we analyzed the data with two types of statistics (frequentist and estimation statistics) to evaluate significance as well as quantitatively express size effects and its precision. In bold bars or continuous curves, we show the data analyzed with frequentist statistics. The statistical tests, exact *n* values, *p* values, and degrees of freedom are described in detail in Table 1, in each figure and figure legend. In brief, a Student’s *t* test was used when a comparison between two independent groups was needed. For multiple groups, data were analyzed using a one-way ANOVA followed by a Tukey’s *post hoc* test. Data from several time points were analyzed using a one-way ANOVA for repeated measures followed by a Dunnett’s *post hoc* test; or a two-way ANOVA for repeated measures, followed by a Sidak’s or Tukey’s *post hoc* test; *p *<* *0.05 was considered statistically significant.

**Table 1:** Statistical table

Figure	Panel	Distribution	Statistical test (frequentist)	Statistical value and *p* value	Panel	Effect size and CI (estimation statistics)
1	*D*	Normal distribution	Unpaired *t* test	*t* = 0.5000 df = 4 *p* = 0.6433 (CI = 95%)	*E*	0.0169 [95.0%CI −1.2, 1.2] *p* = 0.97
	*F*	Normal distribution	Unpaired *t* test	*t* = 0.7559 df = 4 *p* = 0.4918 (CI = 95%)	*G*	0.0209 [95.0%CI −1.19, 1.26] *p* = 0.841
	*H*	Normal distribution	Unpaired *t* test	*t* = 0.7071 df = 4 *p* = 0.5185 (CI = 95%)	*I*	0.0758 [95.0%CI −1.21, 1.29] *p* = 0.886
2	*C*	Normal distribution	One-way ANOVA + Tukey’s *post hoc* test	*F*_(2,6)_ = 81.85 *p* < 0.0001 (CI = 95%)	*D*	VEH vs LPS = 9.47 [95.0%CI 6.63, 12.4] *p* = 0.0
						VEH vs TNFα = −0.0633 [95.0%CI −1.22, 1.24] *p* = 0.908
	*E*	Normal distribution	One-way ANOVA + Tukey’s *post hoc* test	*F*_(2,6)_ = 79.53 *p* < 0.0001 (CI = 95%)	*F*	VEH vs LPS = 8.55 [95.0%CI 6.44, 11.1] *p* = 0.0
						VEH vs TNFα = 0.563 [95.0%CI −0.756, 1.59] *p* = 0.331
	*G*	Normal distribution	One-way RM ANOVA + Dunnett’s *post hoc* test	*F*_(1.306,9.142)_ = 106.2 *p* < 0.0001 (CI = 95%)		
	*H*	Normal distribution	One-way RM ANOVA + Dunnett’s *post hoc* test	*F*_(1.001,2.003)_ = 31.03 *p* = 0.0306 (CI = 95%)		
	*I*	Normal distribution	One-way RM ANOVA + Dunnett’s *post hoc* test	*F*_(1.254,2.508)_ = 74.99 *p* = 0.0061 (CI = 95%)		
3	*A*	Normal distribution	Two-way RM ANOVA + Sidak’s *post hoc* test	*F*_(2,8)_ = 13.63 *p* = 0.0026 (CI = 95%)		
	*C*	Normal distribution	One-way ANOVA + Tukey’s *post hoc* test	*F*_(2,6)_ = 30.98 *p* = 0.0007 (CI = 95%)	*D*	LPS vs LPS+INDO = −6.33 [95.0%CI −8.3, −4.5] *p* = 0.0006
						LPS vs PGE_2_ = 0.147 [95.0%CI −1.27, 1.33] *p* = 0.811
	*E*	Normal distribution	One-way ANOVA + Tukey’s *post hoc* test	*F*_(2 6)_ = 58.42 *p* = 0.0001 (CI = 95%)	*F*	LPS VS LPS+INDO = −6.99 [95.0%CI −9.53, −5.19] *p* = 0.0
						LPS vs PGE_2_= −0.151 [95.0%CI −1.53, 1.04] *p* = 0.789
4	*A*	Normal distribution	One-way ANOVA + Tukey’s *post hoc* test	*F*_(3,20)_ = 33.97 *p* < 0.0001 (CI = 95%)	*B*	LPS vs LPS+INDO =2.29 [95.0%CI 1.47, 3.66] *p* = 0.0008
	*C*	Normal distribution	Two-way RM ANOVA + Tukey’s *post hoc* test	*F*_(48,144)_ = 1.73 *p* = 0.0067 (CI = 95%)		
	*D*	Normal distribution	Unpaired *t* test	*t* = 2.408 df = 7 *p* = 0.0469 (CI = 95%)	*E*	LPS vs LPS+Pro = 1.66 [95.0%CI 0.575, 2.68] *p* = 0.0162
5	*C*	Normal distribution	Unpaired *t* test	*t* = 9.252 df = 4 *p* = 0.0008 (CI = 95%)	*D*	SHAM+LPS vs CAPS+LPS =−8.11 [95.0%CI −10.2, −6.04] *p* = 0.0088
	*E*	Normal distribution	Unpaired *t* test	*t* = 9.583 df = 4 *p* = 0.0007 (CI = 95%)	*F*	SHAM+LPS vs CAPS+LPS =−9.28 [95.0%CI −14.4, −6.8] *p* = 0.0062
	*G*	Normal distribution	Unpaired *t* test	*t* = 3.658 df = 9 *p* = 0.0052 (CI = 95%)	*H*	SHAM+LPS vs CAPS+LPS = 2.49 [95.0%CI 1.67, 7.37] *p* = 0.003.
6	*C*	Normal distribution	One-way ANOVA + Tukey’s *post hoc* test	*F*_(2,15)_ = 60.39 *p* < 0.0001 (CI = 95%)	*D*	SHAM+LPS vs VLX+LPS = −1.6 [95.0%CI −3.37, −0.235] *p* = 0.0136
						SHAM+LPS vs SLX+LPS = −8.12 [95.0%CI −11.8, −5.38] *p* = 0.0
	*E*	Normal distribution	One-way ANOVA + Tukey’s *post hoc* test	*F*_(2,15)_ = 275.2 *p* < 0.0001 (CI = 95%)	*F*	SHAM+LPS vs VLX+LPS = −8.99 [95.0%CI −13.0, −6.12] *p* = 0.0
						SHAM+LPS vs SLX+LPS = −12.4 [95.0%CI −21.8, −7.79] *p* = 0.0
7	*A*	Normal distribution	One-way ANOVA + Tukey’s *post hoc* test	*F*_(2,15)_ = 54.26 *p* < 0.0001	*B*	SHAM+LPS vs VLX+LPS = −0.879 [95.0%CI −2.49, 0.604] *p* = 0.12
						SHAM+LPS vs SLX+LPS = 3.86 [95.0%CI 2.51, 5.21] *p* = 0.0
	*C*	Normal distribution	One-way ANOVA + Tukey’s *post hoc* test	*F*_(2,9)_ = 1799 *p* < 0.0001 (CI = 95%)	*D*	SHAM+LPS vs VLX+LPS = −0.187 [95.0%CI −1.65, 1.3] *p* = 0.742
						SHAM+LPS vs SLX+LPS = 29.0 [95.0%CI 18.7, 59.1] *p* = 0.0
	*E*	Normal distribution	One-way ANOVA + Tukey’s *post hoc* test	*F*_(2,9)_ = 70.82 *p* < 0.0001 (CI = 95%)	*F*	SHAM+LPS vs VLX+LPS = −0.962 [95.0%CI −1.96, 0.218] *p* = 0.0972
						SHAM+LPS vs SLX+LPS = 5.07 [95.0%CI 3.81, 17.3] *p* = 0.0
8	*C*	Normal distribution	Unpaired *t* test	*t* = 12.33 df = 8 *p* < 0.0001 (CI = 95%)	*D*	LPS ZT2 vs LPS ZT14 = −7.04 [95.0%CI −8.91, −5.26] *p* = 0.0066
	*E*	Normal distribution	Unpaired *t* test	*t* = 12.74 df = 8 *p* < 0.0001 (CI = 95%)	*F*	LPS ZT2 vs LPS ZT14 = −7.27 [95.0%CI −9.42, −5.91] *p* = 0.005
	*G*	Normal distribution	Unpaired *t* test	*t* = 5.772 df = 15 *p* < 0.0001 (CI = 95%)	*H*	LPS ZT2 vs LPS ZT14 = 8.32 [95.0%CI 6.24, 10.8] *p* = 0.0008
	*I*	Normal distribution	Two-way RM ANOVA + Sidak’s *post hoc* test	*F*_(2,8)_ = 1.070 *p* = 0.3873 (CI = 95%)		
9	*C*	Normal distribution	Unpaired *t* test	*t* = 6.067 df = 4 *p* = 0.0037 (CI = 95%)	*D*	Low LPS ZT14 vs High LPS ZT14 = 9.94 [95.0%CI 7.51, 12.6] *p* = 0.0042
	*E*	Normal distribution	Unpaired *t* test	*t* = 15.62 df = 4 *p* < 0.0001 (CI = 95%)	*F*	Low LPS ZT14 vs High LPS ZT14 = 10.7 [95.0%CI 8.23, 13.2] *p* = 0.0
	*G*	Normal distribution	Unpaired *t* test	*t* = 0.1512 df = 4 *p* = 0.8871 (CI = 95%)	*H*	ZT2 vs ZT14 = −0.0985 [95.0%CI −5.42, 1.57] *p* = 0.809
	*J*	Normal distribution	Unpaired *t* test	*t* = 10.27 df = 6 *p* < 0.0001 (CI = 95%)	*J*	LPS CT2 vs LPS CT14 = 6.64 [95.0%CI 5.01, 9.7] *p* = 0.0016
10	*A*	Normal distribution	Unpaired *t* test	*t* = 6.140 df = 9 *p* < 0.0002 (CI = 95%)	*B*	LPS ZT2 vs LPS ZT14 = −3.59 [95.0%CI −6.24, −2.13] *p* = 0.0008
	*C*	Normal distribution	Unpaired *t* test	*t* = 4.135 df = 10 *p* < 0.002 (CI = 95%)	*D*	LPS ZT2 vs LPS ZT14 = −2.2 [95.0%CI −3.76, −1.26] *p* = 0.0
	*E*	Normal distribution	One-way ANOVA + Tukey’s *post hoc* test	*F*_(3,12)_ = 8.659 *p* = 0.0025 (CI = 95%)	*F*	LPS vs LPS+INDO = −0.352 [95.0%CI −1.89, 0.851] *p* = 0.529

One-way or two-way ANOVA, repeated measures (RM), confidence intervals (CI).

For the estimation statistics, we introduced the raw data in https://www.estimationstats.com/ and downloaded the results and graphs (Ho et al., 2019). We did not use the estimation statistics for data that were originally analyzed with one-way or two-way ANOVA for repeated measures.

To measure the effect size, we used the Hedges’ *g* estimate, which is the mean difference between two groups divided by the pooled standard deviation of both groups, corrected for small samples (<20). The effect sizes and confidence intervals (CIs) are reported in detail in Table 1 and in each figure as: effect size (CI width lower bound; upper bound). As a rule of thumb for the interpretation of the Hedges’ *g*, an effect size ≤ 0.2 describes a small effect; an effect size ≤ 0.5 describes a medium effect and an effect size ≤ 0.8 describes a large effect.

When comparing two groups, the Hedges’ *g* between control and test is shown with a Gardner–Altman estimation plot. The raw data from both groups are plotted on the left axes; the mean difference is plotted on a floating axis on the right as a bootstrap sampling distribution. The mean difference is depicted as a dot; the 95%CI is indicated by the ends of the vertical error bar.

When comparing three groups, the Hedges’ *g* for two comparisons against a shared control are shown with a Cumming estimation plot. The raw data are plotted on the upper axes. On the lower axes, mean differences are plotted as bootstrap sampling distributions. Each mean difference is depicted as a dot. Each 95%CI is indicated by the ends of the vertical error bars. For each group, summary measurements (mean ± SD) are shown as gapped lines.

In all cases, 5000 bootstrap samples were taken; the CI is bias-corrected and accelerated. The *p* value(s) reported are the likelihood(s) of observing the effect size(s), if the null hypothesis of zero difference is true. For each permutation *p* value, 5000 reshuffles of the control and test labels were performed.

## Results

### LPS administration activates VSAs and not the CVOs

**Figure 1.**
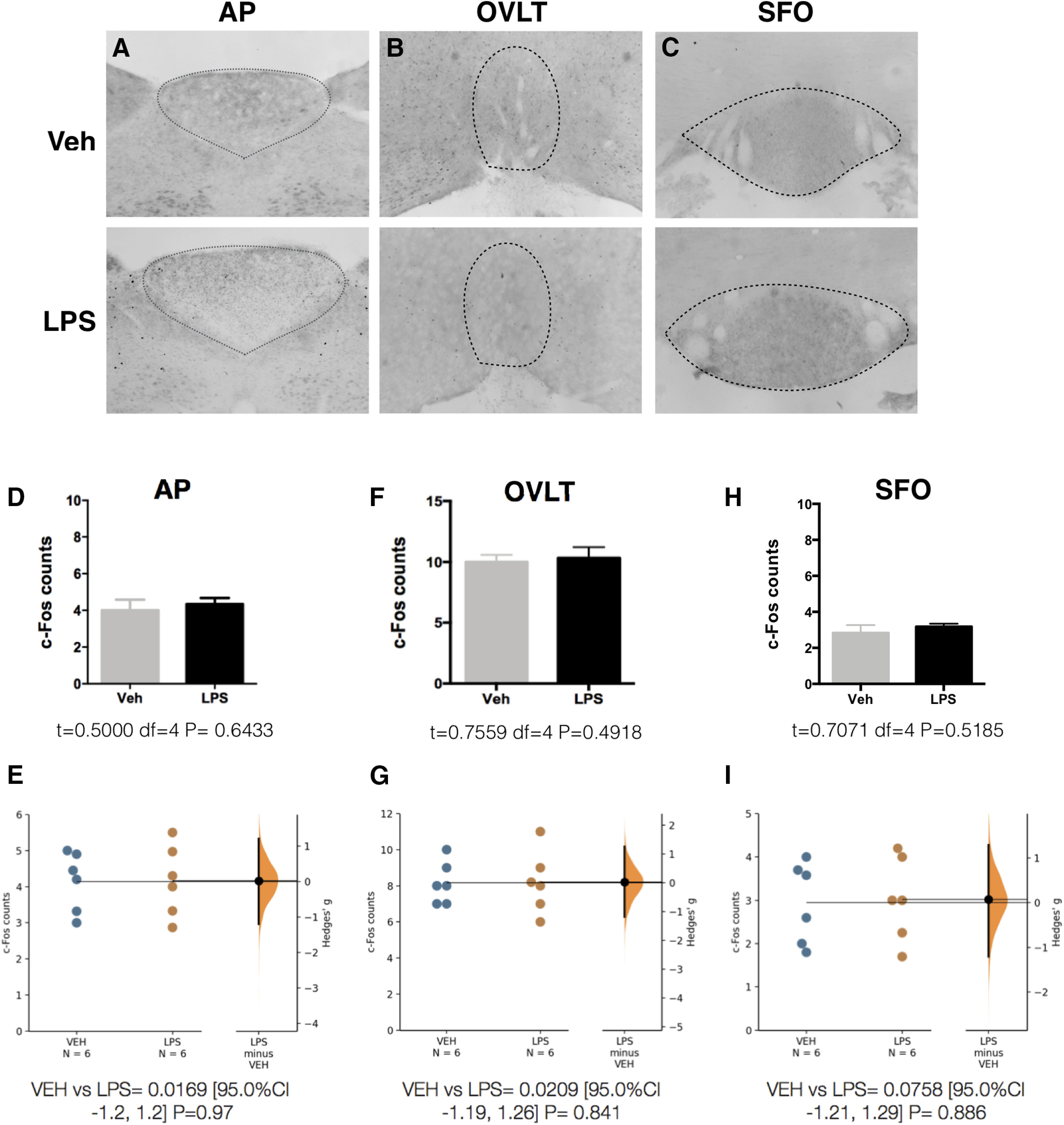
Circulating LPS does not activate the CVOs. Representative pictures showing c-Fos expression in the CVOs: AP (***A***), OVLT (***B***), SFO (***C***) 50 min after Veh or LPS (2 µg/kg) intravenous administration at ZT2. ***D***, ***F***, ***H***, Quantification of c-Fos immunoreactivity for each structure (*n* = 6/group). Data were analyzed with an unpaired two-tailed *t* test. ***E***, ***G***, ***I***, Gardner–Altman estimation plot displaying the raw data from ***D***, ***F***, ***H***, the effect size and the 95%CI.

Male Wistar rats received LPS (2 µg/kg) or Veh intravenously at ZT2 (2 h after the onset of the resting period) and were killed 50 min later, at almost ZT3. We found that under these experimental conditions, LPS administration did not induce c-Fos expression in any of the sensory CVOs previously suggested to participate in LPS sensing (Laflamme and Rivest, 2001; Nakano et al., 2015), indicating that these areas are not able to sense LPS in an early phase (Fig. 1).

**Figure 2.**
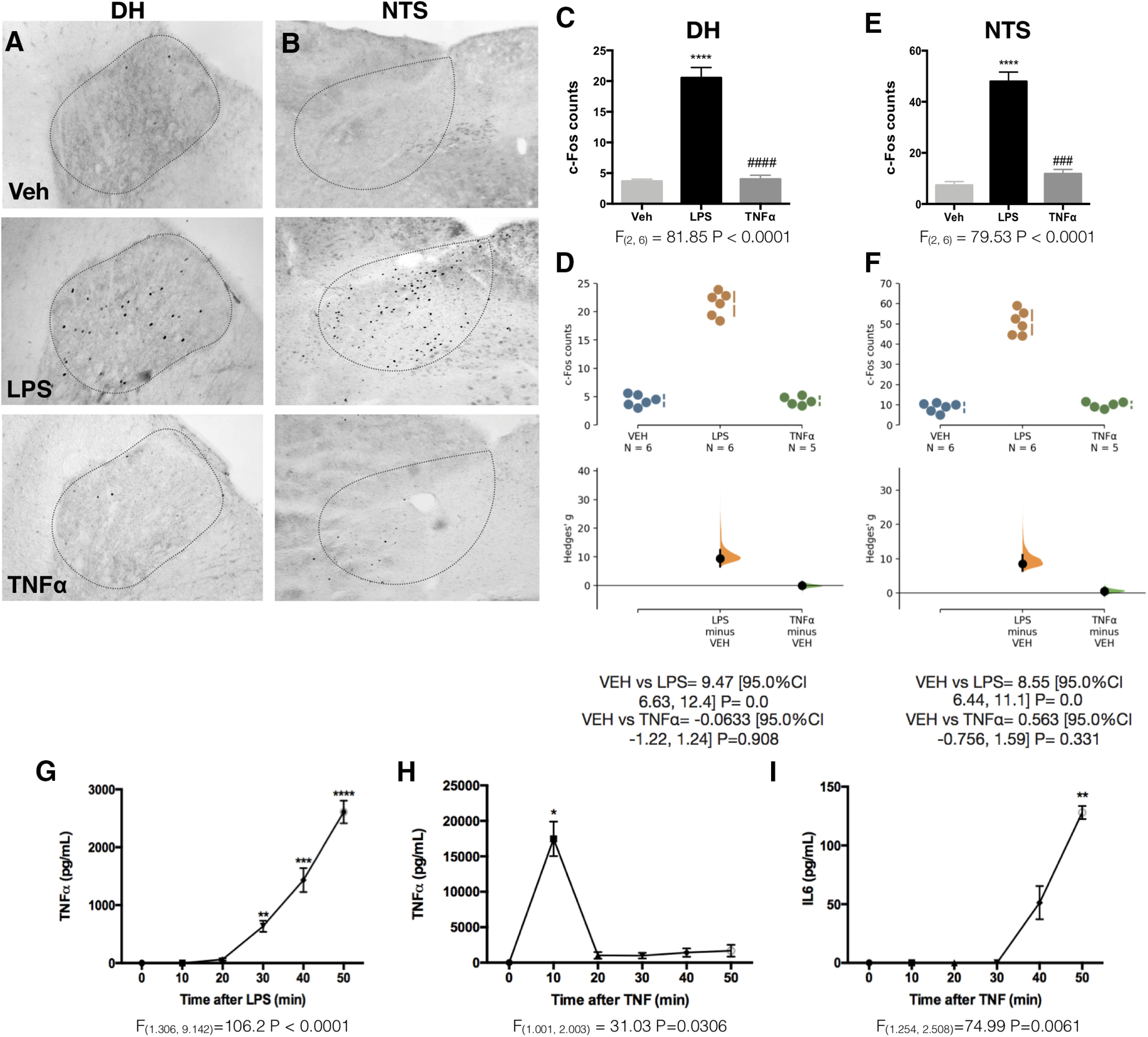
LPS and not TNFα induces neuronal activation in the VSAs. Representative pictures showing c-Fos expression in the VSAs: DH of the spinal cord (***A***) and NTS (***B***) 50 min after Veh, LPS (2 µg/kg), or TNFα (5 µg/kg) intravenous administration at ZT2. ***C***, ***E***, Quantification of c-Fos immunoreactivity for each structure (n=5 -6/group). Data were analyzed with a one-way ANOVA followed by a Tukey's post hoc test; ****p < 0.0001, significance of difference against Veh; ###*p* < 0.001, ####*p* < 0.0001 significance of difference against LPS. ***D***, ***F***, Cumming estimation plot displaying the raw data from ***C***, ***E***, the effect size and the 95%CI. ***G***, Time course of TNFα levels in plasma after the intravenous administration of LPS at ZT2. ***H***, Time course of TNFα concentration in plasma after intravenous administration of TNFα at ZT2. ***I***, Time course of IL-6 concentration in plasma after intravenous administration of TNFα at ZT2. Data in ***G***, ***H***, ***I*** (*n* = 5–6/group) were analyzed with a one-way ANOVA for repeated measures followed by Dunnett’s *post hoc* test; *p < 0.05, **p < 0.01, ***p < 0.001, ****p < 0.0001 significance of difference against time 0.

In the same animals, we evaluated the c-Fos expression in the VSAs (Fig. 2). The dorsal horn (DH) of the spinal cord exclusively receives spinal sensory information, while the NTS located in the brainstem receives both vagal and spinal sensory input. We found that c-Fos expression in these areas was significantly increased after the LPS challenge [DH (Fig. 2*C*,*D*) NTS (Fig. 2*E*,*F*)], suggesting that the VSAs do play a role in the early detection of circulating LPS.

### Prostaglandins (PG) are important mediators for the LPS-induced neuronal activation in the VSAs

As aforementioned, it has been proposed that TNFα can directly signal the inflammatory status of the body to the CNS (Hansen et al., 2001; Dantzer et al., 2008; Zanos et al., 2018). However, detectable traces of TNFα in circulation only appear 20–30 min after LPS administration (Fig. 2*G*). Since c-Fos protein requires 40–60 min to be expressed (Sharp et al., 1991; Hoffman et al., 1993; Lin et al., 2011; Zhong et al., 2014), any c-Fos expression because of TNFα would only be observable after a survival time of 80 min. Considering that we used a survival time of 50 min, neuronal activation cannot be credited to peripheral TNFα. To further rule out the participation of TNFα in the activation of VSAs, we administered recombinant TNFα (5 µg/kg, i.v.) at ZT2. This dose of TNFα was enough to reach a concentration of ∼17 ng/ml in plasma (Fig. 2*H*) and induce the secretion of IL-6 (Fig. 2*I*), proving that this TNFα administration can effectively activate the immune response. Although the concentrations of exogenous TNFα exceeded the LPS-induced TNFα concentrations, this cytokine alone could not induce any neuronal activation in the VSAs within 50 min [DH (Fig. 2*C*,*D*); NTS (Fig. 2*E*,*F*)]. Therefore, we can discard the possibility that TNFα contributes to the early LPS-induced neuronal activation.

**Figure 3.**
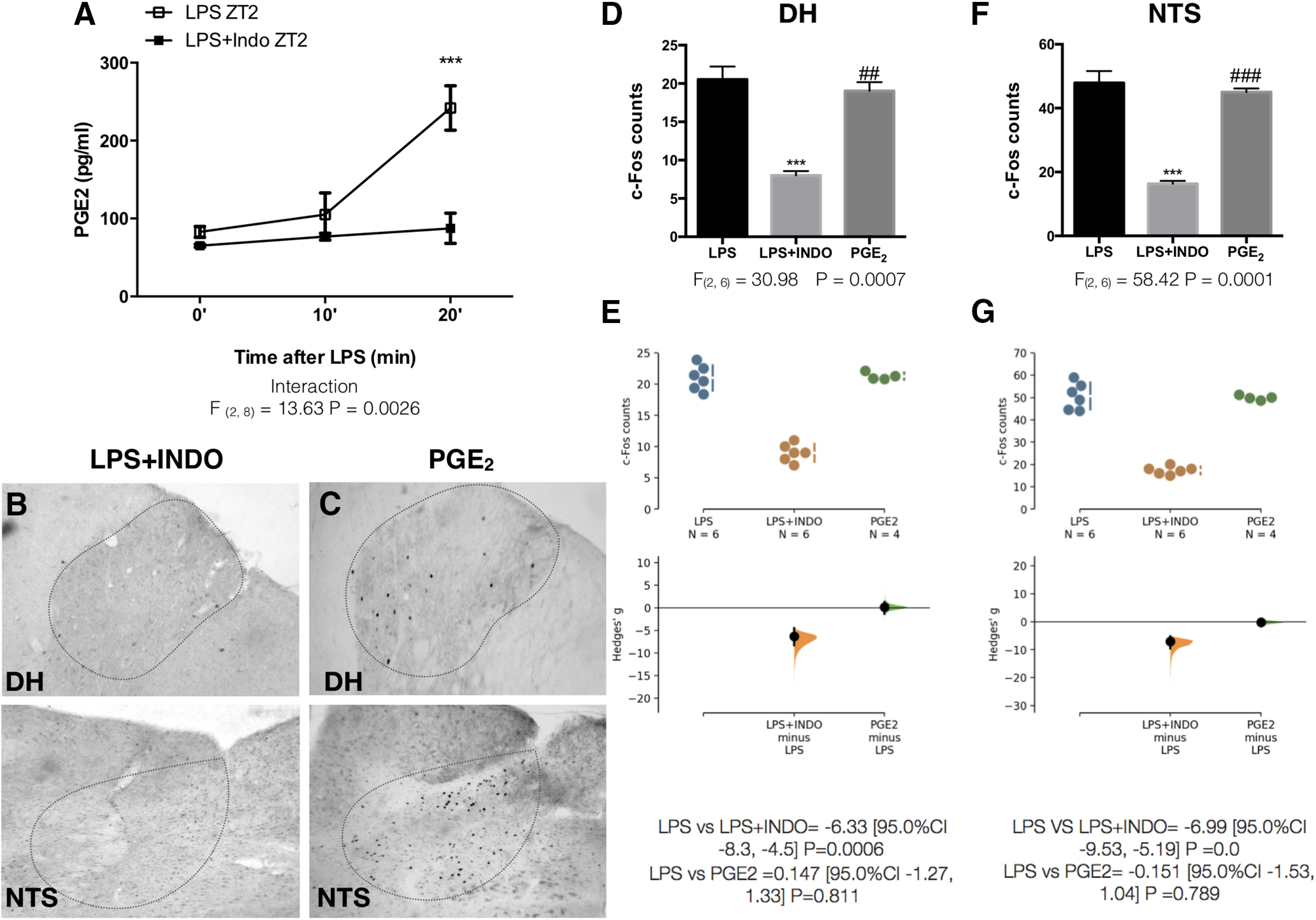
PGs are important mediators for the LPS-induced neuronal activation in the VSAs. ***A***, PGE_2_ levels in plasma at different times after LPS or LPS+Indomethacin (Indo; 10mg/kg) administration (*n* = 6/group). Data were analyzed with a two-way ANOVA for repeated measures followed by a Sidak’s post hoc test; ***p < 0.001 significance of difference between LPS and LPS+Indo. Representative pictures showing c-Fos expression in DH and NTS 50 min after (***B***) LPS+Indo or (***C***) PGE_2_ (100 µg/kg) intravenous administration at ZT2. ***D***, ***F***, Quantification of c-Fos immunoreactivity for each structure (*n* = 4–6 group). Data were analyzed with a one-way ANOVA followed by a Tukey’s *post hoc* test; ***p < 0.001, significance of difference against LPS; ###*p* < 0.001, ##*p* < 0.01 significance of difference against LPS+Indo. ***E***, ***G***, Cumming estimation plot displaying the raw data from ***D***, ***F***, the effect size and the 95%CI.

**Figure 4.**
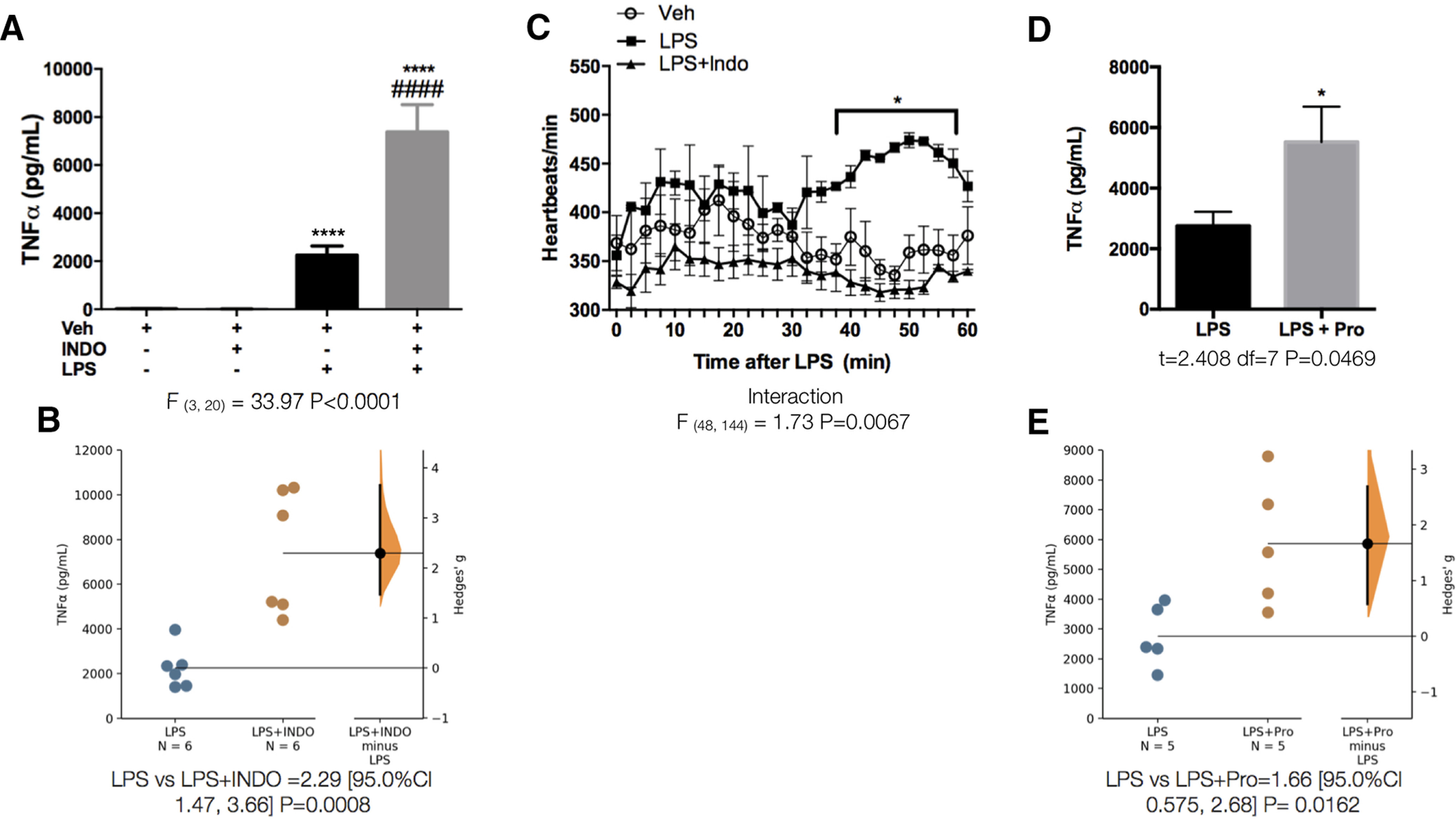
Detection of PG starts an early sympathetic anti-inflammatory reflex. ***A***, TNFα levels in plasma 50 min after treatment with LPS or LPS+Indo (*n* = 6/group). Data were analyzed with a one-way ANOVA followed by a Tukey’s post hoc test; ****p < 0.0001, significance of difference against Veh; ####*p* < 0.0001 significance of difference against LPS. ***B***, Gardner–Altman estimation plot displaying the raw data from A, the effect size and the 95%CI. ***C***, Heart rate measurement during 60 min after Veh, LPS, or LPS+Indo administration (*n* = 3/group). Data were analyzed with a two-way ANOVA for repeated measures followed by a Tukey’s post hoc test; *p < 0.05 significance of difference against LPS. ***D***, TNFα levels in plasma after treatment with LPS or LPS + propranolol (Pro; 1 mg/kg; *n* = 5/group). Data were analyzed with an unpaired two-tailed *t* test; *p < 0.05 significance of difference against LPS. ***E***, Gardner–Altman estimation plot displaying the raw data from ***D***, the effect size and the 95%CI.

PGs play an important role in inflammatory networks and are released 15–20 min after LPS (Li et al., 2006; Fig. 3*A*), which led us to suspect that they could be among the earliest molecules sensed by the VSAs. Blocking PG production by inhibiting COX-1/COX-2 with intravenous indomethacin (10 mg/kg) 30 min before LPS administration at ZT2 (Fig. 3*A*) prevented c-Fos expression in the VSAs [DH (Fig. 3*D*,*E*); NTS (Fig. 3*F*,*G*)], indicating that PGs are important mediators of the LPS-induced neuronal activation in the VSAs. Indomethacin alone did not affect the induction of c-Fos in the VSAs. PGE_2_ is a type of PG that is highly inducible by LPS (Rotondo et al., 1988; Li et al., 2006). When we administered PGE_2_ (100 µg/kg; iv) at ZT2, we observed neuronal activation in the VSAs that resembled that of LPS [DH (Fig. 3*D*,*E*); NTS (Fig. 3*F*,*G*)], suggesting that PGE_2_ could be a likely mediator of LPS. As previously reported (Sacco et al., 1998; Teeling et al., 2010), indomethacin treatment significantly increased LPS-induced TNFα plasma levels (Fig. 4*A*,*B*), suggesting that the sensing of PG by the VSAs contributes to the suppression of TNFα. Furthermore, there was a significant increase in heart rate 30 min after the LPS administration (Fig. 4*C*), probably indicating a general sympathetic activation. The administration of indomethacin effectively prevented this increase in heart rate, suggesting that PG-induced neuronal activation in the VSAs in response to LPS, initiates an early sympathetic reflex that inhibits the secretion of TNFα. To further investigate whether this reflex is sympathetic, we intravenously administered propranolol (Pro), a non-specific antagonist of β-adrenergic receptors, 30 min before the LPS administration. TNFα was increased after the adrenergic blockade (Fig. 4*D*,*E*), strongly suggesting that the efferent arm of the anti-inflammatory reflex comprises sympathetic nerves.

### Liver-spinal afferents start an anti-inflammatory reflex in response to LPS

**Figure 5.**
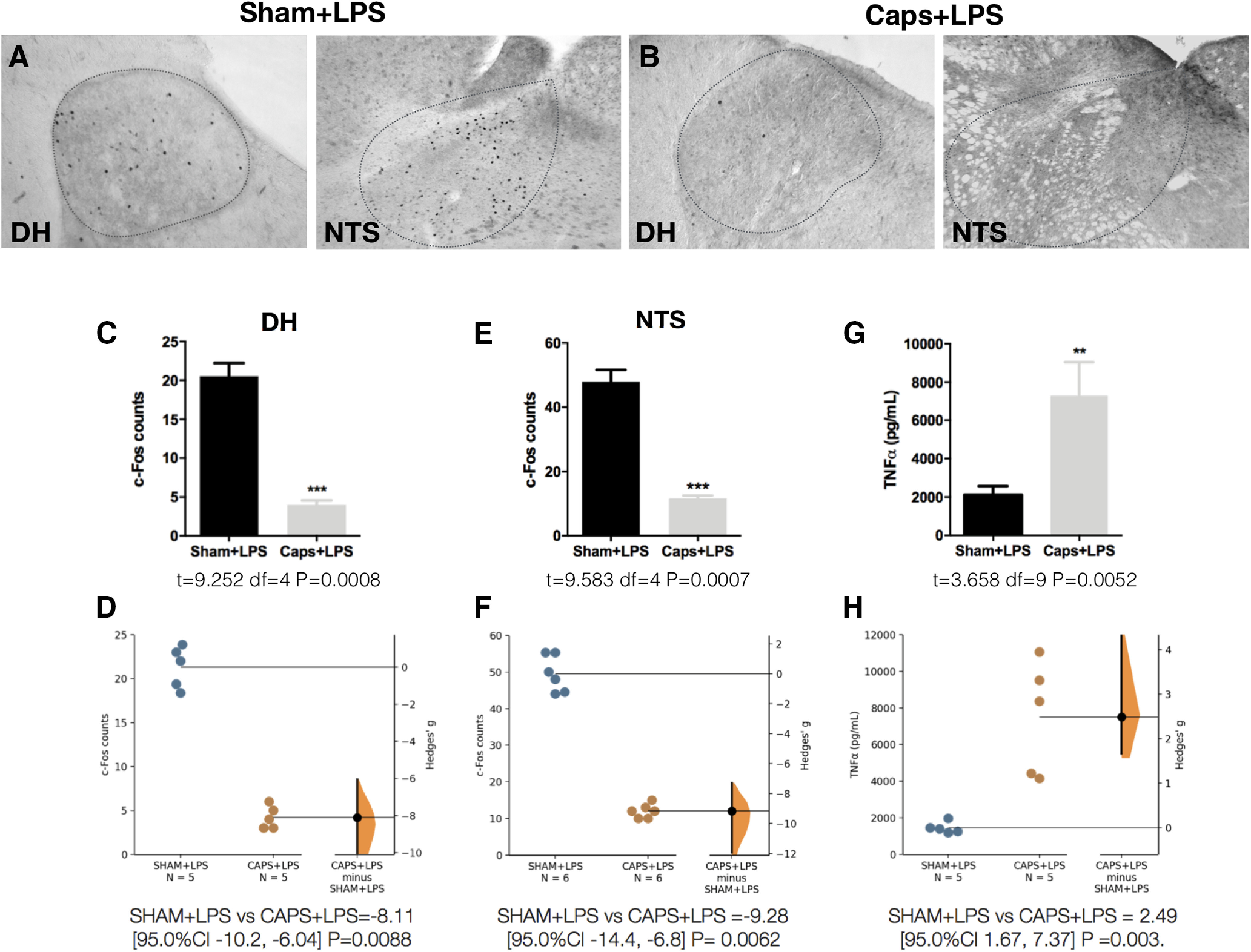
Capsaicin-sensitive afferent nerves engage an anti-inflammatory reflex in response to LPS. Representative pictures of c-Fos expression in DH and NTS 50 min after LPS intravenous administration at ZT2 in (***A***) sham animals (Sham+LPS) or (***B***) animals treated with intraperitoneal capsaicin (Caps+LPS). ***C***, ***E***, Quantification of c-Fos immunoreactivity for each structure (*n* = 5/group). Data were analyzed with an unpaired two-tailed *t* test; ***p < 0.001 significance of difference against Sham+LPS. ***D***, ***F***, Gardner–Altman estimation plot displaying the raw data from ***C***, ***E***, the effect size and the 95%CI. ***G***, TNFα levels in plasma 50 min after LPS treatment. Data were analyzed with an unpaired two-tailed *t* test; **p < 0.01 significance of difference against Sham+LPS. ***H***, Gardner–Altman estimation plot displaying the raw data from ***G***, the effect size and the 95%CI.

To investigate whether visceral sensory nerves transmit circulating immune information to the VSAs, we performed a peritoneal deafferentation with capsaicin. After deafferentation, LPS administration at ZT2 failed to induce c-Fos expression in the VSAs [DH (Fig. 5*C*,*D*); NTS (Fig. 5*E*,*F*)], indicating that the CNS receives circulating immune information via visceral sensory nerves. Compared with the sham animal, deafferented animals displayed increased TNFα levels after the LPS challenge (Fig. 5*G*,*H*), further suggesting that visceral sensory nerves initiate an inhibitory reflex. Basal TNFα levels were not increased in the deafferented animals, ruling out the possibility that capsaicin per se increases the inflammatory response.

Since c-Fos expression is upregulated in NTS and DH after LPS administration and the activation of both VSAs is prevented by peritoneal deafferentation, we needed to elucidate whether spinal and/or vagal sensory nerves were transmitting information necessary for the engagement of the anti-inflammatory reflex. Additionally, we needed to determine the visceral organ from which these afferent nerves originate. In contrast to the spleen, which lacks sensory innervation (Nance and Burns, 1989), the liver has both spinal and vagal sensory innervation (Yi et al., 2010) and plays an important role in the innate immune response (Jirillo et al., 2002). Therefore, we hypothesized that liver afferent nerves were responsible for transmitting peripheral immune information to the CNS.

**Figure 6.**
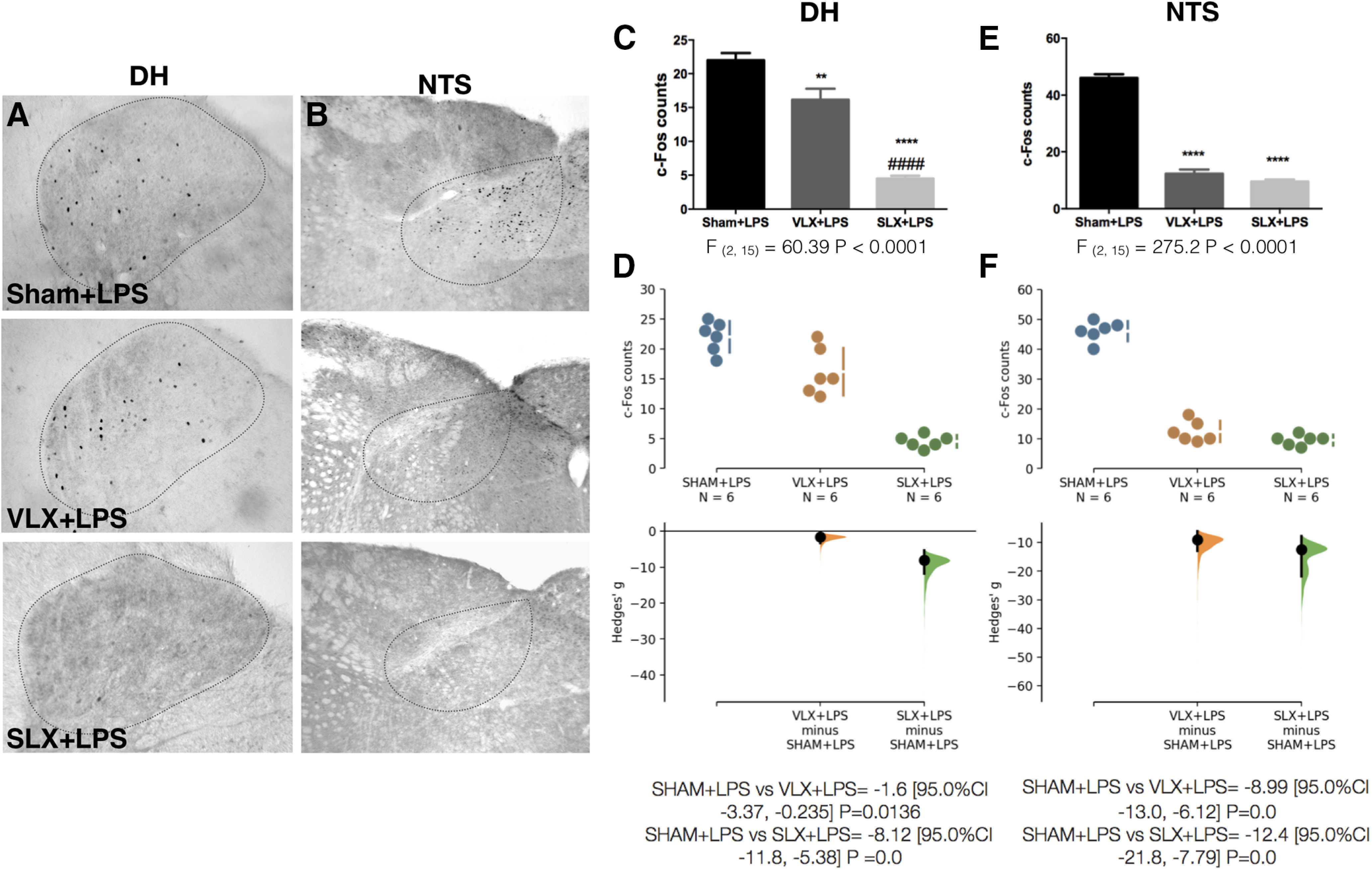
Liver-spinal afferents transmit the inflammatory signal to the VSAs. Representative pictures showing c-Fos expression in (***A***) DH and (***B***) NTS 50 min after LPS intravenous administration at ZT2 in: sham (Sham+LPS), VLX (VLX+LPS), and SLX (SLX+LPS). ***C***, ***E***, Quantification of c-Fos immunoreactivity for each structure (n = 6/group). Data were analyzed with a one-way ANOVA followed by a Tukey's post hoc test; **p < 0.01,****p < 0.0001, significance of difference against sham animal. ***D***, ***F***, Cumming estimation plot displaying the raw data from ***C***, ***E***, the effect size and the 95%CI.

**Figure 7.**
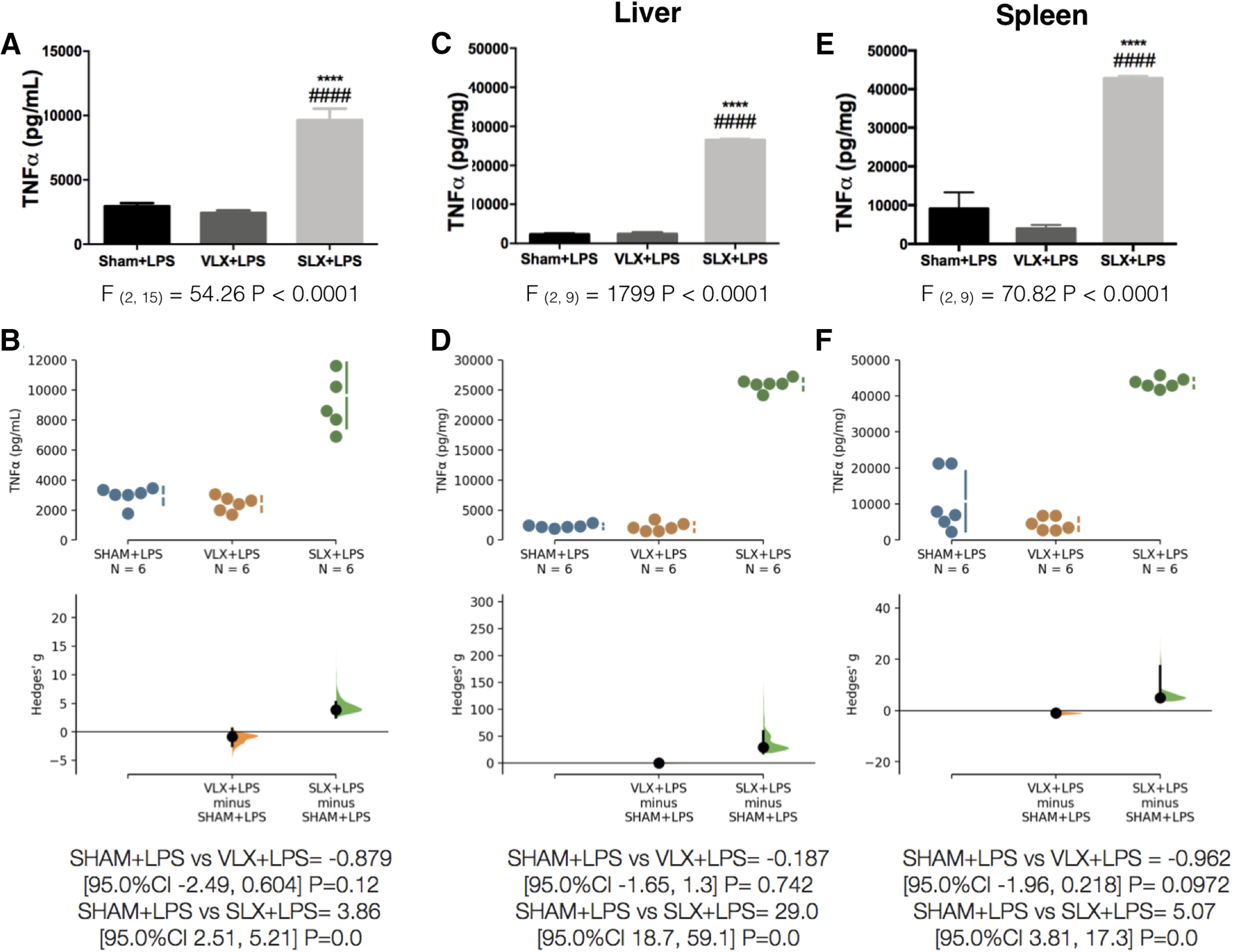
Liver-spinal afferent nerves engage an anti-inflammatory reflex in response to LPS. TNFα quantification in: plasma (***A***), liver (***C***), and spleen (***E***) 50 min after LPS administration. Data from A, C, E were analyzed with a one-way ANOVA followed by a Tukey's post hoc test; ****p < 0.0001, significance of difference against sham animal; ####*p* < 0.0001 significance of difference against VLX animal. ***B***, ***D***, ***F***, Cumming estimation plot displaying the raw data from ***A***, ***C***, ***E***, the effect size and the 95%CI.

To investigate this, we performed SLX or VLX and administered LPS at ZT2. Although c-Fos expression in the NTS was largely suppressed in the VLX animal (Fig. 6*E*,*F*), LPS-induced TNFα plasma concentration remained unchanged (Fig. 7*A*,*B*). These data show that vagal sensory input from the liver is dispensable for the reflex. In contrast, SLX effectively prevented LPS-induced neuronal activation in both VSAs [DH (Fig. 6*C*,*D*); NTS (Fig. 6*E*,*F*)] and significantly increased TNFα levels (Fig. 7*A*,*B*), similar to what happened after peritoneal deafferentation (Fig. 5*G*,*H*). Taken together, liver-spinal afferents are responsible for activating the VSAs and are necessary components of the anti-inflammatory reflex.

Since the liver and spleen are the major sources of TNFα (Huston et al., 2006), the upregulated levels of plasma TNFα seen in the LPS-challenged SLX animals could be accompanied by TNFα content alterations in the liver and spleen. We expected an upregulation of TNFα in the liver because it was the denervated organ, yet to our surprise, we found that TNFα was significantly increased not only in the liver (Fig. 7*C*,*D*), but also in the spleen (Fig. 7*E*,*F*). We can conclude that the liver acts as an LPS sensor, but both liver and spleen serve as the effector organs for TNFα production/release.

### A time-of-day-dependent sensitivity to LPS in the VSAs shapes the TNFα release pattern from liver and spleen

**Figure 8.**
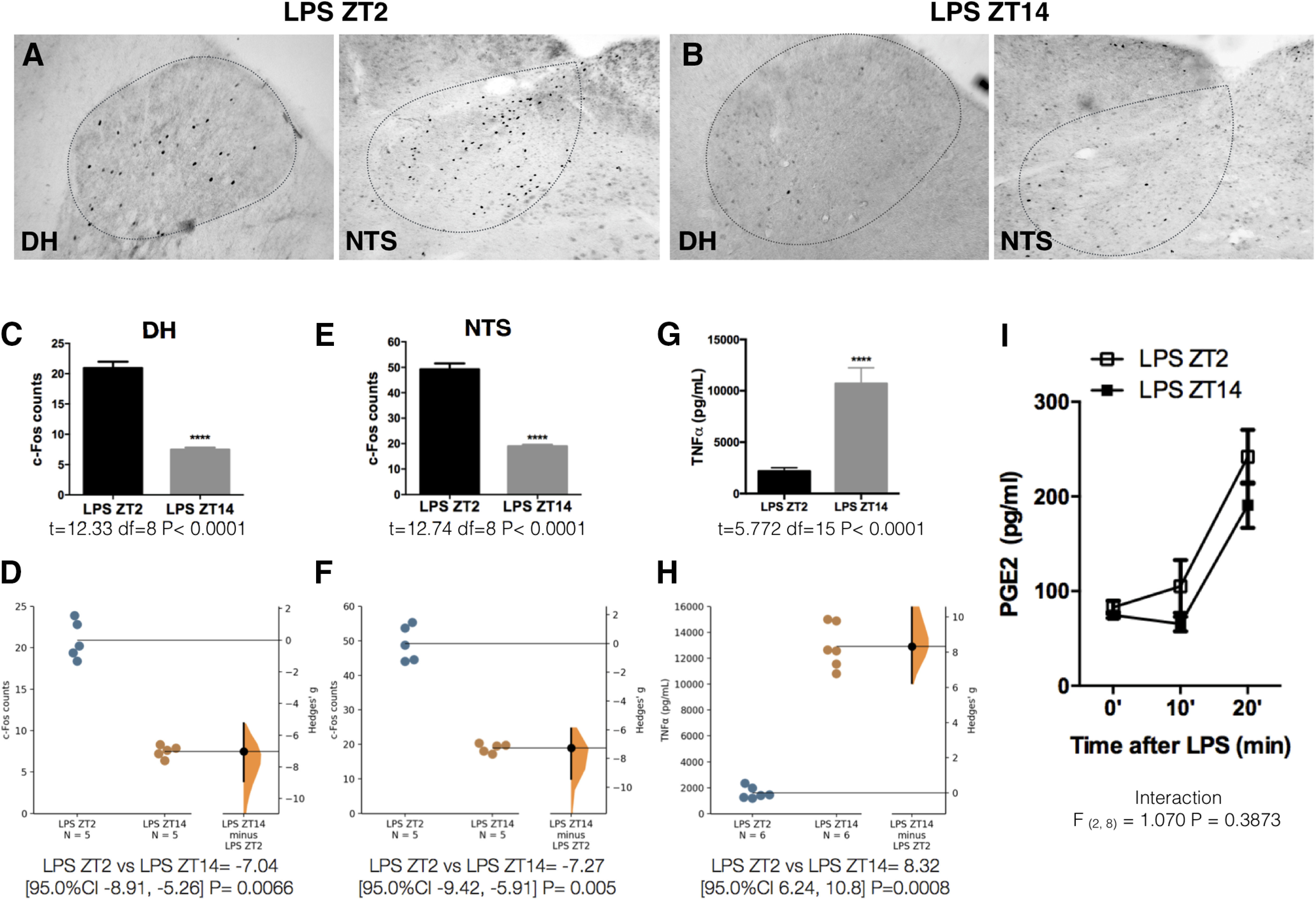
VSAs display a time-of-day-dependent activation in response to LPS. Representative pictures of c-Fos expression in DH and NTS 50 min after LPS intravenous administration at two time points: (***A***) ZT2 (2 h after light onset) or (***B***) ZT14 (2 h after lights off). ***C***, ***E***, Quantification of c-Fos immunoreactivity for each structure (*n* = 5/group). Data were analyzed with an unpaired two-tailed *t* test; ****p < 0.0001, significance of difference between LPS ZT2 and LPS ZT14. ***D***, ***F***, Gardner–Altman estimation plot displaying the raw data from ***C***, ***E***, the effect size and the 95%CI. ***G***, TNFα levels in plasma 50 min after LPS intravenous administration at ZT2 or ZT14 (*n* = 5/group). Data were analyzed with an unpaired two-tailed *t* test; ****p < 0.0001, significance of difference between LPS ZT2 and LPS ZT14. ***H***, Gardner–Altman estimation plot displaying the raw data from ***G***, the effect size and the 95%CI. ***I***, PGE_2_ levels in plasma after LPS intravenous administration at ZT2 or ZT4 (*n* = 5/group). Data were analyzed with a two-way ANOVA for repeated measures followed by a Sidak’s *post hoc* test.

**Figure 9.**
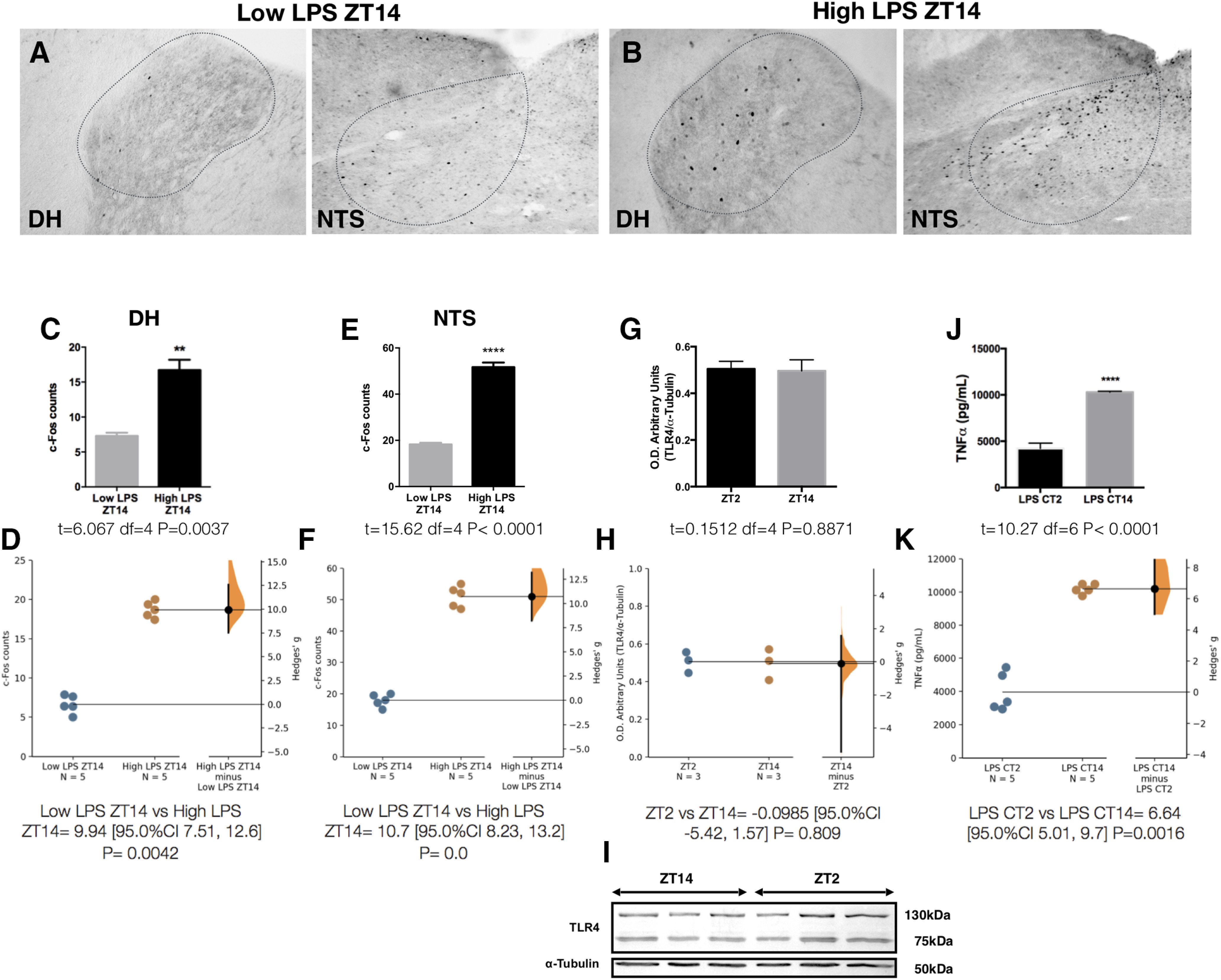
VSAs display a time-of-day-dependent sensitivity to circulating LPS. Representative pictures of c-Fos expression in DH and NTS 50 min after (***A***) low LPS (2 µg/kg) or (***B***) high LPS (100 µg/kg) at ZT14. ***C***, ***E***, Quantification of c-Fos immunoreactivity for each structure (*n* = 5/group). Data were analyzed with an unpaired two-tailed *t* test; **p < 0.01, ****p < 0.0001 significance of difference between low LPS and high LPS. ***D***, ***F***, Gardner–Altman estimation plot displaying the raw data from ***C***, ***E***, the effect size and the 95%CI. ***G***, Quantification of Western blotting for TLR4 relative expression at ZT2 and ZT14 in intact animals (*n* = 3/group). Data were analyzed with an unpaired two-tailed *t* test. ***H***, Gardner–Altman estimation plot displaying the raw data from ***G***, the effect size and the 95%CI. ***I***, Representative image of the TLR4 Western blotting. ***J***, TNFα levels in plasma 50 min after LPS intravenous administration at CT2 or CT14 (*n* = 5/group). Data were analyzed with an unpaired two-tailed *t* test; ****p < 0.0001, significance of difference between LPS ZT2 and LPS ZT14. ***K***, Gardner–Altman estimation plot displaying the raw data from ***J***, the effect size and the 95%CI.

Another important aspect in the central control of inflammation is that the magnitude of the LPS-induced TNFα response depends on the time of the day, being significantly higher during the active period of the animal (Guerrero-Vargas et al., 2014; Fig. 8*G*,*H*). Considering our data, we hypothesized that a time-of-day-dependent sensing of LPS by the VSAs could account for the circadian variations in TNFα plasma concentration. To investigate this, we administered LPS at two different time points, 2 h after light onset (ZT2) and 2 h after light offset (ZT14). We found that when administered at ZT2, LPS induces a significantly higher c-Fos expression in both VSAs compared with ZT14 [DH (Fig. 8*C*,*D*); NTS (Fig. 8*E*,*F*)]. We hypothesized that PG levels are upregulated at ZT2, contributing to the higher VSAs activation at this point. However, PGE_2_ plasma concentration at ZT2 and ZT14 did not differ (Fig. 8*I*), suggesting an increased sensitivity to LPS/PG in the VSAs at ZT2. Administering a higher dose of LPS (100 µg/kg) at ZT14 induced a neuronal activation like the one seen at ZT2 [DH (Fig. 9*C*,*D*); NTS (Fig. 9*E*,*F*)], demonstrating that a time-dependent sensitivity to LPS/PG promotes the different neuronal activation in the VSAs.

**Figure 10.**
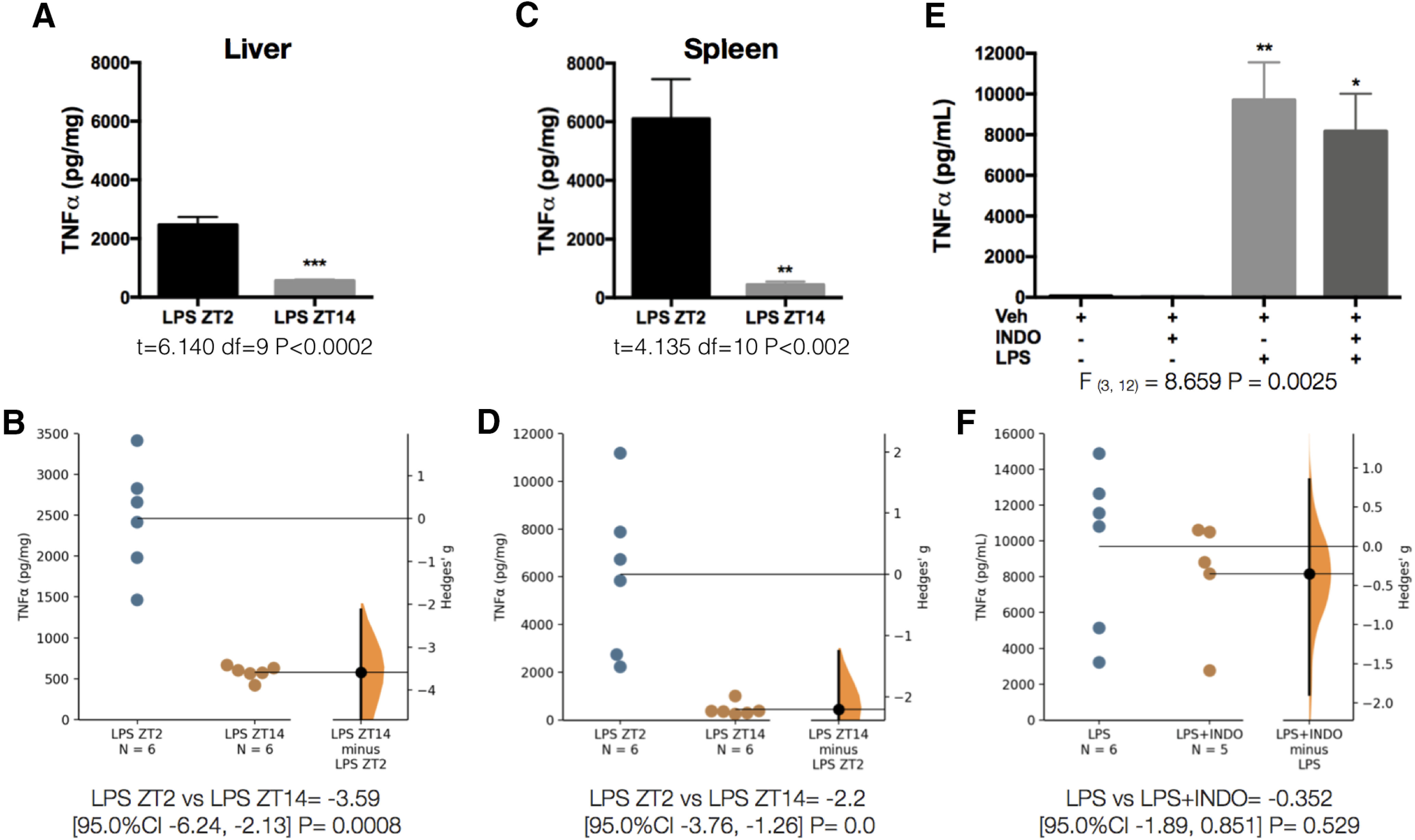
A time-of-day-dependent TNFα release pattern from liver and spleen shapes the rhythm of plasma TNFα. TNFα levels in liver (***A***) and spleen (***C***) 50 min after LPS intravenous administration at ZT2 or ZT14. Data were analyzed with an unpaired two-tailed *t* test (*n* = 5/group); **p < 0.01, ***p < 0.001, significance of difference between LPS ZT2 and LPS ZT14. ***B***, ***D***, Gardner–Altman estimation plot displaying the raw data from ***A***, ***C***, the effect size and the 95%CI. ***E***, TNFα levels in plasma 50 min after LPS or LPS+Indo administration at ZT14. Data were analyzed with a one-way ANOVA followed by a Tukey’s *post hoc* test; *p < 0.05, **p < 0.01 significance of difference against Veh. ***F***, Gardner–Altman estimation plot displaying the raw data from ***E***, the effect size and the 95%CI.

To further test our hypothesis that a time-of-day-dependent sensing of LPS by the VSAs could account for the circadian variations in TNFα plasma concentration, we needed to evaluate other factors that could potentially contribute to the TNFα rhythm. One such factor is the diurnal variation in the expression of TLR4, therefore, we measured the expression of this receptor in the liver of intact animals at ZT2 and ZT14. We did not find a difference between TLR4 expression at these two time points (Fig. 9*G–I*). Another possibility is that TNFα rhythm depends on light entrainment, hence the cytokine was measured under constant dark conditions. The TNFα rhythm persisted even in the absence of light (Fig. 9*J*,*K*), strengthening the view that the inflammatory response to LPS is indeed circadian regulated. Lastly, we reasoned that the circadian rhythm in plasma TNFα could be because of an oscillatory TNFα production by spleen and liver in response to LPS. We found that 50 min after LPS administration at ZT2, the TNFα content in the liver (Fig. 10*A*,*B*) and spleen (Fig. 10*C*,*D*) was elevated, but nearly undetectable when LPS was administered at ZT14. This pattern in TNFα levels is opposite to the one observed in plasma (Fig. 8*G*,*H*), where TNFα is low at ZT2 and high at ZT14.

The administration of indomethacin has a time-of-day-dependent ceiling-effect on LPS-induced TNFα, given that this drug was able to further increase TNFα only during ZT2 (Figs. 4*A*,*B*, 10*E*,*F*). This observation further suggests that the anti-inflammatory reflex is engaged only during the resting period and limits the maximal TNFα response to the active period.

We propose that the time-of-day-dependent gating of the incoming inflammatory signal in the VSAs engages an anti-inflammatory reflex to promote the retention of TNFα within the visceral organs at ZT2; explaining the low plasma TNFα levels at this time point. In contrast, the low LPS/PG sensing in the VSAs during ZT14 does not activate the anti-inflammatory reflex, allowing the organs to freely release most of their TNFα content into circulation. This time-of-day-dependent pattern of TNFα release/retention may explain the circadian rhythm in plasma TNFα response to LPS.

## Discussion

Earlier studies have proposed that CVOs play a role in the sensing of circulating LPS because of the expression of TLR4 in astrocytes (Laflamme and Rivest, 2001; Wuchert et al., 2008; Nakano et al., 2015). Indeed, the induction of c-Fos expression in the CVOs after LPS administration suggests their involvement (Elmquist et al., 1996; Buijs et al., 2008). However, the long survival times (2–6 h) used to evaluate neuronal activation do not exclude the possibility that LPS-induced cytokines, such as TNFα and IL-6, activate the CVOs. To address this issue, we used a shorter survival time (50 min) based on previous findings that indicate a c-Fos expression peak 40–60 min after a stimulus (Sharp et al., 1991; Hoffman et al., 1993; Lin et al., 2011; Zhong et al., 2014). Therefore, we can conclude that the CVOs do not contribute to the early-phase sensing of LPS.

In agreement with our observations, CVOs show a very low basal expression of the mRNA of p55 and p75 TNFα receptors and this expression is upregulated 3–6 h after intraperitoneal administration of LPS or after intravenous administration of TNFα (Nadeau and Rivest, 1999). Consequently, any cytokine signaling to the CVOs most likely occurs in a late-phase of the LPS challenge. This agrees with the proposal that the signaling to the CVOs could account for delayed responses to LPS, such as fever (Blatteis, 1992; Takahashi et al., 1997).

We also tested the possibility that LPS is sensed by the vagus nerve. After cutting the hepatic branch of the vagus, TNFα levels in plasma remained largely unchanged, which indicates that the vagus does not start a reflex to inhibit inflammation. On the other hand, existing literature proposes that proinflammatory cytokines such as IL1β can signal the inflammatory status of the body through the vagus nerve (Ek et al., 1998; Hansen et al., 2001; Zanos et al., 2018). However, as aforementioned, any vagal cytokine sensing may only occur ∼30 min after the LPS challenge, when cytokines appear in circulation, and it is therefore not detected in the present study.

An important factor to consider for the interpretation of our data are the dose of LPS. Other studies have used doses of LPS 50–500 times higher than the dose used here. We are aware that according to the dose and the administration route, other effects can be and have been observed. A previous study by our group (Guerrero-Vargas et al., 2014) evaluated two doses of LPS, 100 and 2 µg/kg. The higher dose proved to be lethal under certain experimental conditions and therefore, a dose of 2 µg/kg was chosen for the present study. This dose effectively activates the immune response and allows a careful dissection of the LPS transmission pathway without overwhelming the system.

The data obtained from the experiments where indomethacin was used indicate that LPS-induced PG are largely responsible for the signaling of inflammatory information to the CNS. One important consideration is that the action of indomethacin is not tissue or cell specific. It has been shown that ∼80% of intravenous injected LPS accumulates in the liver within the first hours after its administration (Jirillo et al., 2002). This visceral organ has an important population of resident macrophages, the Kupffer cells, which could immediately detect the LPS and start producing early proinflammatory molecules such as PG and later cytokines (Li et al., 2006). We hypothesize that indomethacin mainly prevents the production of PG by the liver, however, we have not proven it. PG can also be produced within the CNS (Saper and Breder, 1992), but they need a signal to be synthesized. As previously mentioned, LPS minimally crosses the BBB (Banks and Robinson, 2010), and we have shown that LPS does not activate the CVOs, the areas where PG most likely are synthesized and exert major effects (Takahashi et al., 1997). Therefore, we believe that the production of PG by the liver is the most relevant for the initiation of the anti-inflammatory reflex.

### The liver-spinal axis

We demonstrate the existence of a circuit comprising two crucial aspects. The first element involves the liver-spinal visceral afferents, herein termed as the liver-spinal axis, which is responsible for alerting the CNS about circulating LPS by sensing PG. The fact that the liver is involved in the early transmission of circulating immune information can be expected, since via its portal system, where blood from the gut is received, the liver is the first organ to get exposed to pathogens or toxins ingested with food intake (Jirillo et al., 2002).

We propose this is a spinal reflex since consistently throughout our different experimental conditions, the decrease in c-Fos expression in the DH of the spinal cord was always accompanied by higher circulating TNFα levels. These data strongly suggest that the higher neuronal activation in the DH seen at ZT2 serves to prevent a high inflammatory process. The same did not happen in the NTS, since the loss of its activation in the VLX condition was not accompanied by an increase in TNFα levels. Spinal afferent nerves are normally associated with sensorimotor reflexes and nociceptive pathways. Our study shows that the spinal cord can use similar pathways to act on the inflammatory process, which is consistent with other studies that have reported the expression of various TLRs, including 4, 3, and 7, in visceral sensory neurons of the DRG (Barajon et al., 2009; Ochoa-Cortes et al., 2010). The circuit we describe may not completely fulfill the definition of a classical spinal reflex, which implies that the circuit should be complete only within the spinal cord. In the present study we show that this reflex also involves areas outside of the spinal cord, particularly the NTS, which is located in the brainstem. As for the circuits involved within the spinal cord, previous retrograde viral tracing studies following the sympathetic branch of the liver, showed tracing of the sympathetic motor neurons in the intermediolateral column of the spinal cord (IML), followed by tracing of the neurons in the DH; indicating that such direct spinal-sympathetic reflex is possible (Kalsbeek et al., 2004).

The use of capsaicin to ablate afferent spinal nerves close to the portal system could also compromise vagal afferents, which opens the question of whether vagal afferents also play a role in the early signaling of LPS. This possibility could also potentially explain the loss of NTS activation after the SLX denervation. Nonetheless, removing the hepatic branch of the vagus nerve did not prevent c-Fos activation in both VSAs as observed after the SLX denervation. Moreover, vagal denervation had no effect on TNFα levels, which is strong evidence to conclude that spinal and not vagal visceral afferents are responsible for the anti-inflammatory reflex observed in the present study.

### Time-of-day-dependent anti-inflammatory reflex

The second crucial aspect of the described pathway is its rhythmicity. We show that the sensitivity of the VSAs to incoming inflammatory input is time-of-day-dependent, whereby the VSAs receive this information only during the resting period; preventing a strong inflammatory process at this point. In contrast, when the VSAs do not receive this information during the active period, the anti-inflammatory process is not initiated; resulting in higher circulating TNFα levels. The fact that pain also shows a circadian rhythm (Pickard, 1987; Konecka and Sroczynska, 1998; Zhang et al., 2012) is in agreement with our data, indicating that the circadian system may render visceral sensory neurons more or less sensitive to stimuli, including inflammatory signals.

The rhythm in the TNFα response to LPS has been previously observed and its circadian regulation is evidenced by the fact that lesioning the central clock, the suprachiasmatic nucleus, results in an exacerbated inflammatory response to LPS (Guerrero-Vargas et al., 2014). In addition, desynchronization also promotes an exacerbated inflammatory response to LPS (Guerrero-Vargas et al., 2015). Our data propose a mechanism for the circadian control of the TNFα response. A time-of-day-dependent activity of the liver-spinal axis promotes the retention of TNFα in the liver and spleen during the resting period, promoting a lower plasma TNFα level at this point.

One factor that could also influence the rhythmicity of TNFα is the endogenous rhythm of corticosterone. We have previously shown that the corticosterone levels at ZT14 are considerably higher than those observed at ZT2 (Kalsbeek et al., 1996). However, corticosterone is known to have anti-inflammatory effects (Smoak and Cidlowski, 2004; Baschant and Tuckermann, 2010). Therefore, the high corticosterone levels observed at ZT14 do not agree with the high levels of TNFα observed at this time point. Similarly, the low levels of corticosterone observed at ZT2 should promote higher TNFα levels, but that is not the case. For this reason, it is unlikely that the circadian rhythm in corticosterone contributes to the TNFα rhythm.

TNFα is synthesized as transmembrane pro-TNFα and is cleaved from the cell membrane by the metalloprotease ADAM17 (also known as TNFα converting enzyme; Bell et al., 2007). Observations in primary cultures of cortical neurons showed that noradrenaline can decrease the enzymatic activity of ADAM17 (Madrigal et al., 2017). Our data suggest that the anti-inflammatory reflex is sympathetic because of its impairment by propranolol. Therefore, we hypothesize that the function of ADAM17 is hampered by noradrenaline released by the anti-inflammatory reflex during the resting period, thus accounting for the accumulation of TNFα in liver and spleen.

Since the probability of encountering a pathogen is higher during the active period than during the resting period, the time-of-day-dependent activation of the liver-spinal axis could be an adaptive mechanism to narrow the time-window of the inflammatory response to when it is most likely needed.

In conclusion, the present study describes a novel pathway by which the anti-inflammatory reflex is engaged after an LPS challenge: the liver-spinal axis. The early detection of circulating LPS is conducted by spinal visceral afferents and not by the CVOs nor vagal visceral afferents. In addition, this sensing is largely dependent on the presence of PGs. Finally, we prove that the sensing of LPS by the liver-spinal axis is time-of-day dependent and shapes the circadian rhythm of TNFα.

## Synthesis

Reviewing Editor: Rae Silver, Columbia University Decisions are customarily a result of the Reviewing Editor and the peer reviewers coming together and discussing their recommendations until a consensus is reached. When revisions are invited, a fact-based synthesis statement explaining their decision and outlining what is needed to prepare a revision will be listed below. Note: If this manuscript was transferred from JNeurosci and a decision was made to accept the manuscript without peer review, a brief statement to this effect will instead be what is listed below.

This is quite an interesting paper, and it addresses a little investigated area of neuro-immune system responses. The authors have responded very clearly and thoroughly responded to the previous reviews, which were themselves extremely thoughtful.

A problem which the authors must tackle is with regard to the presentation for the results and the statistical analyses. As indicated in the instructions to authors, this research is a good candidate to include estimation statistics upon revision. More information about estimation statistics can be found here: https://doi.org/10.1523/ENEURO.0259-19.2019

A minor, administrative point is that I had some problems with the material as uploaded. The DOC version apparently had no figures. The PDF separated the figures from the (not-numbered) figure legends. There appears to be a visual abstract, though this too is not labelled as such. That said, I was able to align the material (for this review).

## Author Response

Dear Dr. Silver,

We thank you and the reviewing editor for provisionally accepting our manuscript. As we were transferred from J. Neuroscience to eNeuro, we were unaware of the policy of eNeuro to stimulate the use of estimation statistics as another (additional) way to present the data of the study.

After examining this, for us new approach, we appreciate the new visual way to present data. However, since we hardly found papers in eNeuro that used this format, we also thought to support the editorial effort by presenting our results in the classical way together with estimate statistics. In this way, we think our readers may better appreciate better the approach of eNeuro for new presentation and valorization of the presented data.

Please find our manuscript in which we have included the estimation statistics and appropriate changes in the manuscript indicated in red.

With kind regards

Ruud M Buijs PhD
